# *Mycobacterium tuberculosis* complex Lineage 8 causes drug resistant pulmonary tuberculosis also in South Kivu, Eastern Democratic Republic of Congo

**DOI:** 10.3389/fcimb.2026.1918557

**Published:** 2026-10-01

**Authors:** Bertin C. Bisimwa, Esto Bahizire, Isabel Cuella Martin, Leen Rigouts, Wim Mulders, François Hakizayezu, Valery Byela, Hosee Niyompano, Eliane Kamanzi, Freddy Birembano, Steven Callens, Jean Claude S. Ngabonziza, Conor J. Meehan, Bouke C. de Jong

**Affiliations:** 1 Centre for tropical diseases and global health, Université Catholique de Bukavu, Bukavu, Democratic Republic of Congo; 2 Faculty of medicine and health sciences, Ghent University, Ghent, Belgium; 3 Mycobacteriology unit, Institute of tropical medicine, Antwerp, Belgium; 4 Institut Supérieur des Techniques Médicales de Bukavu, Bukavu, Democratic Republic of Congo; 5 Department of microbiology, University of Nairobi, Nairobi, Kenya; 6 Centre de Recherche en Sciences Naturelles, Lwirho, Democratic Republic of Congo; 7 National Reference Laboratory Division, Rwanda Biomedical Centre, Kigali, Rwanda; 8 National Tuberculosis Program, Kinshasa, Democratic Republic of Congo; 9 Department of internal medicine, Ghent University Hospital, Ghent, Belgium; 10 Research, Innovation and Data Science Division, Rwanda Biomedical Centre, Kigali, Rwanda; 11 Department of Clinical Biology, University of Rwanda, Kigali, Rwanda; 12 Centre for Systems Health and Integrated Metabolic Research, Nottingham Trent University, Nottingham, United Kingdom

**Keywords:** African Great Lakes region, drug-resistant tuberculosis, Lineage 8, *M. tuberculosis*, South Kivu

## Abstract

**Background:**

Lineage 8 (L8) occupies a unique phylogenetic position as a sister clade to all other known *Mycobacterium tuberculosis* complex (MTBC) lineages. Although the initial identification of L8 strains was limited to Rwanda and Uganda, the complete geographical distribution of this lineage remains undetermined due to limited genomic surveillance across eastern and central Africa.

**Methods:**

We performed whole genome sequencing (WGS) on MTBC isolates from rifampicin-resistant tuberculosis (TB) patients’ sputum in Bukavu, South Kivu province, eastern Democratic Republic of Congo (DRC). Lineage assignment was performed using TBProfiler with classification based on single nucleotide polymporphisms (SNPs). Phylogenetic reconstruction was conducted using maximum likelihood analysis against publicly available L8 reference genomes from Rwanda and Uganda. Drug-resistance profiles were determined through both genotypic prediction from WGS data and phenotypic drug-susceptibility testing. Clinical and epidemiologic data were extracted from programmatic records.

**Results:**

We identified three clinical isolates belonging to MTBC L8 among fifteen sequenced isolates collected in Bukavu between January and December 2023 enriched for rifampicin resistant TB as determined by Xpert Ultra. Phylogenetic analysis confirmed placement within the L8 clade while revealing unique SNP profiles distinct from previously published Rwandan and Ugandan L8 strains by 16–20 to >200 SNPs, respectively, consistent with local diversification rather than recent cross-border importation. Ancestral genomic features characteristic of L8 were preserved, including the intact *cobF* region and complete *pks8* gene. Drug-resistance prediction from genomic data indicated multidrug-resistant and pre-extensively-resistant TB.

**Conclusions:**

This report extends the known geographic distribution of MTBC L8 to eastern DRC, supporting the hypothesis that the African Great Lakes region harbors under-characterized ancestral diversity of the tubercle bacillus. Systematic genomic surveillance across the DRC-Uganda-Rwanda-Burundi corridor, including rifampicin-susceptible isolates, is needed to determine whether L8 represents a rare relict lineage or an under-detected endemic variant circulating through regional transmission networks.

## Introduction

Tuberculosis (TB) remains the leading cause of death from a single infectious agent globally, responsible for an estimated 1.3 million deaths among HIV-negative individuals and an additional 167,000 among people living with HIV in 2024 (World Health Organization, 2025a). The disease is caused by members of the *Mycobacterium tuberculosis* complex (MTBC), a group of closely related mycobacterial species and lineages that have coevolved with human populations over thousands of years (Comas et al., 2014). Genomic analyses have revealed that the MTBC is structured into at least ten human-adapted lineages (L1 through L10), each with characteristic geographic distributions reflecting the migration history of their sympatric host populations (Gagneux, 2018; Coscolla et al., 2021; Goig et al., 2025). Understanding the phylogenetic diversity and geographic range of these lineages is fundamental to TB control, as lineage-specific differences in virulence, transmissibility, immunogenicity, and propensity for drug resistance have been described with potential implications for vaccine development, diagnostics performance, and treatment outcomes (Meehan et al., 2019; Getahun et al., 2025).

Africa is the only continent where all known MTBC lineages are found, strongly supporting the hypothesis that the complex originated on the continent before expanding globally following human migrations (NGabonziza et al., 2020; Couvin et al., 2025; Danso et al., 2025). In 2020, Ngabonziza and colleagues described a previously unknown lineage, designated lineage 8 (L8), from a clinical *M. tuberculosis* isolate obtained from a patient with multidrug-resistant tuberculosis (MDR-TB) in Rwanda and one isolate from Uganda (NGabonziza et al., 2020), which both shared the same *rpoB* Asp435Tyr mutation. Phylogenetic reconstruction demonstrated that L8 occupies a singular evolutionary position as a sister clade to all other known MTBC lineages, branching between the smooth tubercle bacilli (*Mycobacterium canettii*) and the most recent common ancestor of the classical MTBC (NGabonziza et al., 2020; Orgeur et al., 2024). Critically, comparative genomic analysis revealed that L8 retains ancestral genomic regions that have been deleted in all other known MTBC lineages, including an intact *cobF* gene region involved in cobalamin (vitamin B12) biosynthesis and a complete *pks8* gene, which is interrupted by a frameshift mutation in the common ancestor of L1–L7 (NGabonziza et al., 2020). These findings provided molecular evidence that the divergence of L8 preceded key genome reduction events in the evolutionary trajectory of the MTBC.

The discovery of L8 followed the earlier identification of lineage 7 (L7), which is largely restricted to Ethiopia and the Horn of Africa, and preceded the description of lineage 9 (L9), an *M. africanum*-affiliated lineage found in Djibouti and Somalia (Firdessa et al., 2013; Coscolla et al., 2021). More recently, a tenth lineage (L10), also affiliated with *M. africanum*, was identified in central Africa, sister to L6 and L9 (Guyeux et al., 2024).

Together, these discoveries indicate that previously unrecognized MTBC diversity persists in parts of Africa where genomic surveillance has been limited. The East African Rift has been proposed as an early centre of MTBC diversification, supported by the localized distribution of *M. canettii* in the Horn of Africa and the identification of L8 in the African Great Lakes region.

The eastern DRC, and South Kivu province in particular, sits at the geographic and demographic intersection of the African Great Lakes region. Bukavu, the provincial capital, is separated from Cyangugu (Rusizi) in Rwanda by only a bridge, and the province shares borders with Burundi to the south. Decades of armed conflict, population displacement, and cross-border migration have created conditions favoring both TB transmission and the mixing of mycobacterial populations circulating in neighboring countries (World Health Organization, 2023a). Despite this epidemiologic significance, molecular characterization of MTBC strains circulating in eastern DRC has been extremely limited, and no systematic genome-based surveillance has been published from this region.

Defining the geographic distribution of L8 has implications beyond documenting a new lineage occurrence. Because L8 diverged before several genome reduction events that shaped the evolution of the classical MTBC, identifying additional isolates provides an opportunity to refine current models of MTBC evolution and reconstruct the historical dispersal of ancestral lineages across the African Great Lakes region. Characterizing the resistance profiles of these isolates also helps determine whether drug resistance in L8 reflects recent local acquisition or broader transmission within this lineage.

Given the phylogeographic restriction of L8, and the absence of genomic data from eastern DRC, it remained unknown whether this deeply branching lineage extends beyond Rwanda and Uganda. We report the first identification and phylogenetic characterization of L8 *M. tuberculosis* isolates from clinical specimens collected in South Kivu province, eastern DRC. We describe the genomic characteristics of these isolates, compare their drug-resistance-associated mutation profiles with co-circulating lineages, present the clinical and epidemiologic features of affected patients, and discuss the implications for TB molecular surveillance and regional collaboration in the Great Lakes corridor.

## Methods

### Study setting and population

Routine programmatic rifampicin-resistant (RR-) TB sputum specimens from South Kivu, confirmed on Xpert MTB/RIF Ultra, were shipped to the Rwanda Biomedical Centre (RBC) for phenotypic drug-susceptibility testing (pDST), through a specific Material Transfer Agreement signed between the RBC and the DRC National TB Program to support advanced diagnostics for RR-TB patients in Eastern DRC. Ethical approval for further strain characterization was obtained from the institutional ethical committee of the Université Catholique de Bukavu, ITM IRB and RBC ethics (part of SOFI InnoR3TB). All participants provided written informed consent.

### Sample collection and mycobacterial culture

In RBC, after NALC-NaOH based decontamination, sediments were inoculated on both solid (in-house prepared Löwenstein-Jensen) and liquid (BBL MGIT, Becton Dickinson, USA) media. Presence of MTBC was confirmed by Bioline TB Ag MPT64. pDST was performed using the MGIT AST/SIRE System (Becton Dickinson, Sparks, MD, USA).

### Genomic DNA extraction and whole genome sequencing

Colonies grown on solid culture were suspended in 150 µL 0.5 M Tris EDTA buffer and heat inactivated, and subsequently shipped to the Institute of Tropical Medicine (ITM) in Antwerp, Belgium, where genomic DNA was extracted using an in-house optimized protocol (Nguyen et al., 2020). Whole genome sequencing (WGS) was performed by CD Genomics using the Illumina NovaSeq paired end 150 bp platform, generating approximately 0.5 GB of raw data corresponding to about 80 to 100× genome coverage. Non-MTBC reads were removed from FASTQ datasets using Centrifuge v1.0.3, retaining reads classified as MTBC (Kim et al., 2016). Samples with < 90% of reads assigned to MTBC were removed. Reads were mapped to the H37Rv reference genome using MTBseq with default parameters, including a minimum allele frequency threshold of 75 percent for variant calling (Lempens et al., 2018). All retained samples had > 90% of reads mapping to the reference, >90% of the reference bases being covered and a minimum depth of 30x.

### Lineage and drug resistance profile prediction

Drug-resistance profiles were inferred with TB Profiler v6 using the TBDB curated mutation database (branch WHO; commit: 29483bdc) and interpreted according to the WHO catalogue of resistance associated mutations (Version 2) (World Health Organization, 2023b). Lineage was also assigned using TB Profiler in the same run.

The assignment of L8 isolates was further confirmed using a phylogenetic approach. A SNP alignment of all isolates classified as L8 by TB Profiler was created using the MTBseq Amend functionality with default parameters (sites retained if unambiguous in 95% of samples; SNPs removed if two occur within 12 bases of each other). Repetitive regions such as PE/PPE genes were masked from this alignment, as were SNPs in common resistance-related genes (e.g. *rpoB*, *katG* etc.). This SNP alignment was then input to RAxML NG v1.2.0 under a GTR plus gamma model that accounted for constant sites in the genome, along with a Stamatakis ascertainment bias correction (Phelan et al., 2019) and 200 bootstrap replicates. The resulting tree was visualized using iTOL with midpoint rooting.

### Regions of difference and ancestral genomic feature analysis

The presence or absence of key regions of difference was assessed by mapping reads to the relevant genomic coordinates in the H37Rv reference and the published L8-genome assembly. Specifically, the integrity of the *cobF* gene region (Rv0943c–Rv0944) and the *pks8* locus was evaluated by assessing read coverage and variant calls in the corresponding coordinates, as described by Ngabonziza et al. (NGabonziza et al., 2020).

### Statistical analysis

This study was designed as a descriptive genomic characterization study. Data were summarized using descriptive measures only, and no inferential statistical analyses were performed because the objective was to characterize the genomic, clinical, and epidemiological features of the identified isolates rather than test predefined hypotheses.

## Results

### Clinical and epidemiologic characteristics

Ninety-six sputum specimens from 58 patients, comprising RR-TB patients, diagnostic specimens from retreatment patients and follow-up specimens from patients already on treatment, were collected from different TB diagnostics centers in South Kivu, and sent for culture and pDST at the RBC, Kigali. Among them, 26 (27,1%) were culture positive, the rest were negative or contaminated. All 26 positive cultures underwent pDST, and among them, 16 (61,5%) were MDR-TB. Of the 26 culture positive isolates, 15 (57,7%) RR-TB isolates were whole genome sequenced, with three L8 strains being identified by TB-profiler. pDST results were concordant with the resistance profiles predicted by WGS for all drugs tested (**Table 1**).

**Table 1:** Epidemiological information and drug-resistance-associated mutations in lineage 8 isolates.

Isolate ID	Location	Patient history	Patient occupation	pDST results (1^st^ line and 2^nd^ line)	Drugs and related gene(s) with observed mutations						
					RIF/*rpoB*	INH/*katG*	INH-ETH/*inhA*	EMB/*embB*	SM/*rpsL*	FQs/*gyrA*	PZA/*pncA*
DRC1 (CT2023-02824)	DRC - Kitutu	Retreatment	Farmer	RIF R, INH R, BDQ S, LVX S, CFZ S, LNZ S	His445Leu	Ser315Thr	Ser94Ala	Met306Leu	–	–	Ser164*
DRC2 (CT2023-02808	DRC - Bukavu	New	School teacher	RIF R, INH R, BDQ S, LVX S, CFZ S, LNZ S	His445Leu	Ser315Thr	Ser94Ala	Glu405Asp	–	Asp94Gly	Asp63Ala
DRC3 (CT2024-00313)	DRC - Walungu	Retreatment	Mining	RIF R, INH R, BDQ S, LVX S, CFZ S, LNZ S	His445Leu	Ser315Thr	Ser94Ala	–	–	–	Asp63Ala
Rwanda1 (CT2021-02222)	Rwanda - Rulindo	New	Farmer		Asp435Tyr	–	Ser94Ala	–	–	–	–
Rwanda2 (CT2018-01172)	Rwanda - unknown	New	Farmer		Asp435Tyr	–	Ser94Ala	–	–	–	–
Uganda (SRR1173284)	Uganda - unknown	Unknown	Unknown		Asp435Tyr	Ser315Thr	Ser94Ala	–	–	–	Leu85Arg; Pro54Gln

RIF (rifampicin), INH (isoniazid), ETH (ethionamide), EMB (ethambutol), SM (streptomycin), FQs (fluoroquinolones), PZA (pyrazinamide), BDQ (bedaquiline), LVX (levofloxacin), CFZ (clofazimine), LNZ (linezolid), R (resistant), S (sensitive), * indicates a translation termination (stop) codon.

The *M. tuberculosis* L8 South Kivu strains were isolated from three male patients with a median age of 39 years (IQR: 24-47). One patient had no previous TB treatment history, while two had failed prior RR-TB treatment (**Table 1**). All three patients tested HIV negative. The sputum of the DRC1 isolate was collected at the health centre of the Communauté Baptiste au Centre de l’Afrique (CBCA) Nyamungo, in the health district of Kadutu, one of the 3 health districts of Bukavu city. The DRC2 isolate was collected at the Kitutu General Hospital, in the health district of Kitutu and the DRC3 isolate at the Walungu General Hospital, in the health district of Walungu.

One L8-infected patient, a school teacher, resided in Bukavu city, which lies on the southwestern shore of lake Kivu, directly west of the Rwandan town of Rusizi. The second, a farmer, resided in Kitutu (212 Km away from Bukavu) and the third patient, who was involved in mining activities, resided in Walungu (45 Km away from Bukavu) (**Table 1**, **Figure 1**). We could not identify, through the National TB Program registries, any social or epidemiologic link between the three patients.

**Figure 1:**
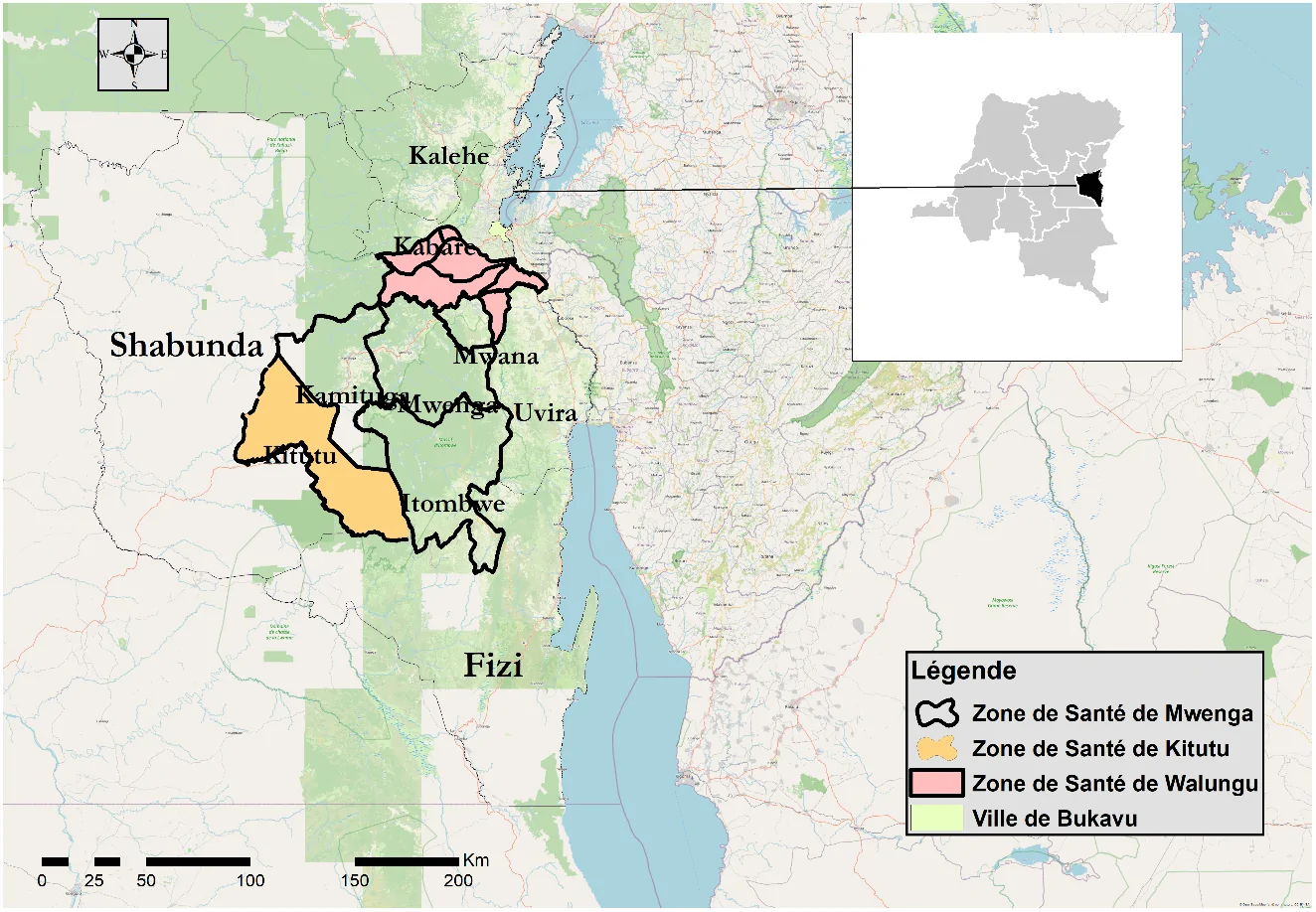
Mapping of the 3 health zones in the South Kivu province, where the samples were collected. Orange (Kitutu health zone), pink (Walungu health zone), green (Kadutu health zone, in Bukavu city).

### Phylogenetic reconstruction

The three South Kivu isolates identified as L8 by TB Profiler were analyzed with one publicly available L8 genome from Uganda and two from Rwanda. The three South Kivu isolates clustered together in the maximum likelihood phylogeny. Pairwise SNP differences were 18 to 26 between the South Kivu and Ugandan isolates and 199 to 209 between the South Kivu and Rwandan isolates, based on 231 variable sites in the alignment. Among the three South Kivu isolates, pairwise differences were 9, 11, and 12 SNPs. Using a published *Mycobacterium tuberculosis* substitution rate of 0.3 to 0.5 SNPs per genome per year, these differences corresponded to an extrapolated timescale of approximately 18 to 40 years (Bryant et al., 2013; Menardo et al., 2019). This estimate represents an extrapolation from published substitution rates rather than a formal molecular dating analysis performed in the present study (**Figure 2**).

**Figure 2:**
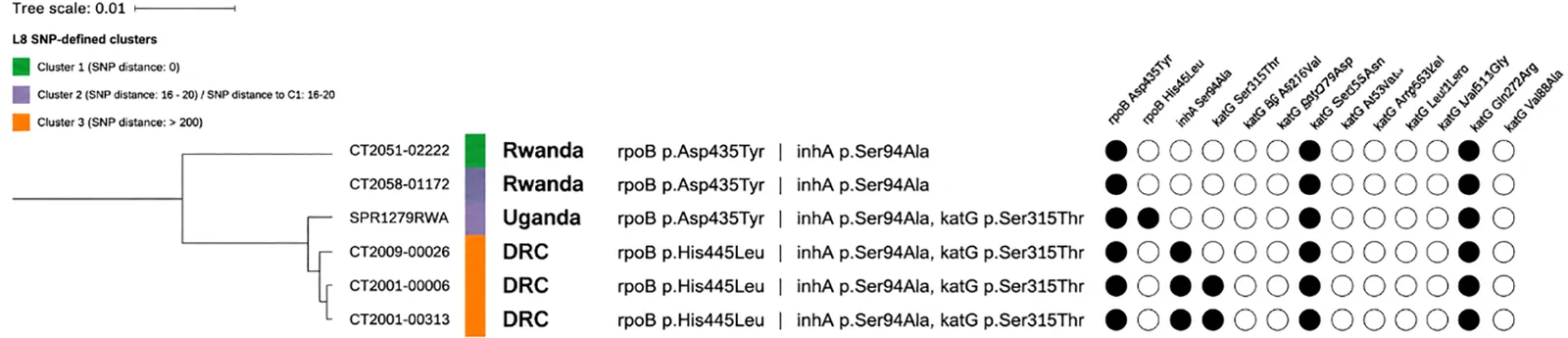
Maximum likelihood phylogeny of MTBC genomes including South Kivu lineage 8 isolates. Scale bar indicates substitutions per variable site. L8 isolates from South Kivu are labeled “DRC” (Democratic Republic of the Congo) in orange. The phylogeny is midpoint rooted. SNP (single nucleotide polymorphism); MDR-TB (multi drug-resistant tuberculosis); Pre XDR-TB (Pre extensively drug-resistant tuberculosis).

### Preservation of ancestral genomic features

Analysis of regions of difference confirmed that the South Kivu L8 isolates retained the ancestral *cobF* gene region, which is deleted in all L1-L7 and L9-L10 strains and in animal-adapted lineages (**Table 2**). The *cobF* region was present with uniform read coverage (region median depth coverage: 144, 104, 99 respectively) and no evidence of disruption. Similarly, the *pks8* coding sequence was intact without the 1-nucleotide frameshift deletion that interrupts pks8/17 in the H37Rv reference and other known modern MTBC lineages. These findings were fully consistent with the phylogenetic position of the original L8 strains and confirmed the similar ancestral character of the South Kivu L8 isolates.

**Table 2:** Status of ancestral genomic regions in South Kivu L8 isolates compared with reference strains.

Genomic region	L8 South Kivu	L8 Rwanda (reference)	L8 Uganda	L2–L4 (H37Rv)	*M. canettii*
*cobF* region	Intact	Intact	Intact	Deleted	Intact
*pks8* gene	Complete	Complete	Complete	Interrupted (frameshift)	Complete

### Drug resistance profile

Genotypic drug-resistance prediction from WGS data revealed that all three South Kivu isolates were harboring *rpoB* His445Leu and *katG* Ser315Thr and mutations. Two isolates were harboring an additional *inhA* ser94Ala mutation, and one isolate a *gyrA* Asp94Gly mutation (**Figure 2**, **Table 1**). No mixed resistance profiles suggestive of heteroresistance were detected among the analyzed isolates.

## Discussion

We report the first identification of *M. tuberculosis* L8 in the eastern DRC among patients with drug resistant pulmonary TB, extending the known geographic range of this deeply branching lineage beyond Rwanda and Uganda. Phylogenetic analysis confirmed that the South Kivu isolates belong to L8 and are related to those from Rwanda and Uganda. Since they are <1000 SNPs from the previously designated L8 strains this confirms their placement in the lineage as based on cut-offs defined by Coscolla et al (Coscolla and Gagneux, 2014). Although all are MDR, the South Kivu L8 shared a different *rpoB* mutation from the one shared by the “Rwanda-Uganda” L8. The isolates retained the ancestral genomic features that define L8, including the intact *cobF* region and complete *pks8* gene.

The identification of L8 in South Kivu is consistent with previous reports from Rwanda and Uganda and extends the known geographic distribution of this lineage within the African Great Lakes region. Although based on only three isolates, these findings support existing evidence that uncommon MTBC lineages are present in this region and highlight the need for broader genomic surveillance to define their frequency and distribution (NGabonziza et al., 2020; Coscolla et al., 2021).

The initial description of L8 from one Rwandan and one Ugandan isolates left open the question of whether this lineage represented a geographically restricted relict or whether it had remained largely undetected because genomic surveillance in the region has been limited. The identification of three genetically distinct L8 isolates in eastern DRC is consistent with the latter possibility and extends the known geographic range of this lineage. Although these data do not establish long term circulation or transmission within the African Great Lakes region, they support the need for larger population based genomic studies to determine the prevalence, evolutionary history, and transmission patterns of L8.

The phylogenetic position of L8 as a sister clade to all other MTBC lineages, combined with the retention of ancestral genomic regions lost in the common ancestor of L1-L7 and L9-L10, positions this lineage as a critical reference point for understanding the early evolutionary events that shaped the MTBC (NGabonziza et al., 2020).

South Kivu province occupies a unique epidemiologic position at the intersection of DRC, Rwanda, and Burundi, three countries linked by intensive cross-border migration, displacement, and trade (World Health Organization, 2023b). Bukavu and the Rwandan city of Rusizi function essentially as a binational urban corridor, with thousands of daily border crossings. The eastern DRC has experienced decades of armed conflict resulting in massive population movements, which are known drivers of TB transmission and genetic mixing of pathogen populations. In this context, the identification of L8 in South Kivu is not surprising but rather expected. The SNP distance between the South Kivu and Rwandan L8 isolates suggests that this could be a single recent introduction. It reflects a lineage with an established regional presence, consistent with sustained transmission across the Great Lakes area over years to decades.

The original L8 strains from Rwanda and Uganda were both multidrug-resistant, a finding that initially raised concerns about whether L8 might be inherently associated with drug resistance (NGabonziza et al., 2020). However, this association was potentially a surveillance artifact, as those strains were identified through a targeted MDR-TB diagnostic trial (DIAMA) that preferentially sampled drug-resistant isolates. Similarly, our screen in South Kivu was enriched for drug resistance and retreatment patients, with a low recovery rate on primary isolation, precluding unbiased estimates of the prevalence of L8 in the region. Comparison of drug resistance-associated mutations across lineages revealed different resistance patterns between L8 and co-circulating strains among *rpoB* gene mutations. While all South Kivu L8 isolates shared the His445Leu *rpoB* mutation, ser450Leu is globally the most common *rpoB* mutation in South Kivu (Bisimwa et al., 2021).

As the L8 isolates we identified showed different drug-resistance patterns, we expect also wildtype L8 is circulating in Central Africa. More systematic genetic analysis of rifampicin-susceptible MTBC isolates, such as in a nationwide drug resistance survey with sufficient representation of Eastern DRC, is required to investigate this hypothesis.

From an evolutionary perspective, the ancestral genomic features retained by L8, particularly the intact *cobF* region involved in cobalamin synthesis and the complete *pks8* polyketide synthase gene, raise questions about potential differences in metabolic capacity, cell wall composition, and host-pathogen interaction compared with the modern lineages (NGabonziza et al., 2020). While *cobF* loss in modern lineages represents a well-characterized evolutionary adaptation associated with host dependency for vitamin B12, the functional significance of *pks8* retention in L8 remains unclear. Experimental validation is needed to determine whether these genomic differences translate into measurable phenotypic distinctions in metabolic capacity, cell wall composition, additional non-human reservoirs, or virulence. Analysis of a large collection of Vietnamese *M. tuberculosis* isolates demonstrated that targets of selection are lineage-specific and involve diverse biological functions, including dormancy (Rv0080), zinc homeostasis (zur), and virulence, particularly components of the ESX-1 secretion system (Silcocks et al., 2026).

The identification of L8 in eastern DRC has several implications for TB surveillance and control. First, it underscores the need to learn about MTBC diversity and potential relatedness with virulence and pathogenicity to gain new insights. Similarly, it would be good to study minimal inhibitory concentrations for anti-TB drugs, to investigate lineage-specific drug-susceptibility patterns, similar to L1 or L6 being less or more susceptible to pretomanid, for instance (Rojas et al., 2025; Kumar et al., 2026). A nationwide drug-resistance survey is currently underway in the Democratic Republic of the Congo. However, in South Kivu, only one of the 34 health zones has been included to date, primarily due to prevailing security constraints. Second, the cross-border distribution of L8 across DRC, Rwanda, and Uganda supports regional approaches to TB molecular surveillance. The Great Lakes region, with its shared epidemiology, population mobility, and confirmed sharing of unique pathogen lineages, would benefit from a coordinated genomic surveillance network that transcends national boundaries. The DRC notified over 270,000 TB cases in 2024, yet almost none have been subjected to WGS-based lineage classification.

From a clinical perspective, the identification of L8 does not currently justify changes to TB diagnosis or treatment. The South Kivu isolates were detected through routine WHO endorsed molecular diagnostic algorithms, indicating that currently recommended assays remain suitable for detecting TB caused by this lineage (World Health Organization, 2025b). WGS added value by placing these isolates within an uncommon MTBC lineage while simultaneously providing high resolution characterization of drug resistance associated mutations. As sequencing capacity expands, integration of genomic data into national surveillance programs, consistent with current WHO recommendations (FIND-WHO, 2023; World Health Organization, 2025b), will improve detection of uncommon MTBC lineages, support investigations of cross border transmission, and refine our understanding of TB population structure.

This study has several limitations. The number of L8 isolates identified is small, limiting prevalence estimates and statistical comparisons with other lineages and precluding definitive conclusions about clinical phenotype or transmission dynamics. Isolates were obtained from programmatic diagnostics at three different facilities in South Kivu enriched for patients with complex treatment histories and confirmed resistance, and the proportion of L8 among all TB cases in South Kivu may not be accurately estimated from this convenience sample. Whole genome sequencing was performed only on RR isolates. This sampling strategy introduces a selection bias and does not allow estimation of the prevalence of L8 among all TB cases or assessment of whether drug susceptible L8 strains also circulate in the region. Population based genomic studies that include both drug susceptible and drug resistant isolates will be needed to define the frequency and distribution of L8 in eastern DRC. Contact tracing data were not available, preventing reconstruction of epidemiological chains. Finally, the absence of WGS data from Burundi and from other provinces of DRC limits our ability to define the full geographic extent of L8 circulation.

The phylogenetic relationships observed among the L8 isolates should be interpreted cautiously given the small number of available genomes and the limited number of variable sites in the alignment. The study population was also enriched for RR and MDR-TB, and independently acquired resistance associated mutations may contribute to homoplasy and affect phylogenetic distances. Yenew et al. used ClonalFrameML to account for recombination when examining deeper evolutionary relationships within and around the MTBC clade, illustrating the potential influence of recombination on branch length estimation (Yenew et al., 2023). In the present study, the molecular clock calculation was used only as a broad extrapolation for the closely related South Kivu isolates and was not used to infer the timing or direction of L8 introduction into South Kivu.

The *pks5* status of the L8 isolates was not assessed, limiting our ability to evaluate the timing of the *pks5* fusion event relative to the divergence of L8.

We report the first identification of *M. tuberculosis* L8 in eastern DRC, extending the known geographic distribution of this deeply branching ancestral lineage within the African Great Lakes region. The genetic differences observed between the South Kivu isolates and the previously reported isolates from Rwanda and Uganda suggest that L8 may have circulated in the region for longer than currently recognized. Given the limited number of sequenced isolates, this interpretation should be considered a hypothesis that requires confirmation through larger population based genomic surveillance studies. Our findings indicate that the Great Lakes region may harbour previously unrecognized MTBC diversity and support expanding genomic surveillance in eastern DRC to clarify the distribution, transmission patterns, and evolutionary history of L8.

## Data Availability

The datasets presented in this study can be found in online repositories. The names of the repository/repositories and accession number(s) can be found below: https://www.ebi.ac.uk/ena, ERS29636309 https://www.ebi.ac.uk/ena, ERS29636310 https://www.ebi.ac.uk/ena, ERS29636311.
